# The Tribe Hyrtanellini Allen, 1980 (Ephemeroptera: Ephemerellidae) of Western and Central Asia with Description of a New Species †

**DOI:** 10.3390/insects14010087

**Published:** 2023-01-13

**Authors:** Alexander V. Martynov, Dmitry M. Palatov, Roman J. Godunko

**Affiliations:** 1 National Museum of Natural History, National Academy of Sciences of Ukraine, Bohdan Khmelnytsky Str., 01601 Kyiv, Ukraine; 2 Independent Researcher, 79000 Lviv, Ukraine; 3 Biology Centre of the Czech Academy of Sciences, Institute of Entomology, Branišovská 31, 37005 České Budějovice, Czech Republic; 4 Department of Invertebrate Zoology and Hydrobiology, University of Łódź, Banacha 12/16, 90237 Łódź, Poland

**Keywords:** spiny crawler mayflies, Tajikistan, Iran, type locality, larva, variation, key, distribution, COI

## Abstract

**Simple Summary:**

The larvae of tribe Hyrtanellini Allen, 1980 are very important from the biomonitoring and bioindication point of view. Unfortunately, the immature stage of some Hyrtanellini is difficult to identify as many authors have stressed. Moreover, in the mountainous areas of western and central Asia there is still clearly a significant number of undescribed new species. The present study focuses on the immature stage of the species of Hyrtanellini from Western and Central Asia. A new species of *Serratella leonidi* Martynov & Palatov, **sp. nov.** from Tajikistan is described, and generic placement of *Serratella elissa* Jacobus, Zhou & McCafferty, 2009 from Iran is discussed. Additionally, the phylogenetic reconstruction of Hyrtanellini based on the COI gene is proposed and discussed. Finally, considering the importance of correct identification of larvae for biomonitoring and investigation of aquatic fauna diversity, the key for the immature stage is shaped.

**Abstract:**

A new species, *Serratella leonidi* Martynov & Palatov, **sp. nov.**, is described from Tajikistan based on immature stage. Based on larval material from Iran including the topotypes, *Serratella elissa* Jacobus, Zhou & McCafferty, 2009 is complementary described, and its generic placement is clarified. The delimitation of three genera that are members of the tribe Hyrtanellini Allen, 1980, namely *Serratella* Edmunds, 1959, *Torleya* Lestage, 1917 and *Quatica* Jacobus & McCafferty, 2008 is briefly discussed. The phylogenetic reconstruction of Hyrtanellini based on the COI gene showed the relations of representatives of these genera on the one hand, and distinct delimitation of *Serratella leonidi* **sp. nov.** and *S. elissa* on the other. A list of species from Western and Central Asia attributed to Hyrtanellini, their currently known distribution and a key for the determination of the larvae are proposed.

## 1. Introduction

Spiny crawler mayflies (Ephemerellidae) are not speciose family comprising about 160 extant species known from the Nearctic, Palearctic and Oriental realms [1]. Despite the low number of species within the family, its taxonomical structure remains dynamic. A review by Jacobus and McCafferty [2] significantly ordered the family, but borders between some of the genera, including some genera of the tribe Hyrtanellini Allen, 1980, are still diffused. *Serratella* Edmunds, 1959, *Torleya* Lestage, 1917 and *Quatica* Jacobus & McCafferty, 2008 can be treated as such genera. Within Western and Central Asia, these genera were known with six species [3,4,5,6,7,8,9]. Another genus of Hyrtanellini Allen, 1980 known from these territories is *Teloganopsis* Ulmer, 1939, presented here with four species [5,8,10,11,12]. Recently, Ding et al. [13] described Chinese *Serratella* and reported six valid species, including one new species from Sichuan and Yunnan Provinces. The authors also briefly discussed the importance of the future systematic studies of the genera *Serratella*, *Ephemerella* and *Torleya*.

Despite such a small number of Asian species of the tribe Hyrtanellini, the data on the distribution, variability and ecological preferences of its representatives are fragmentary. Populations of only some species from these areas of Asia were the object of molecular investigations [14,15,16].

The current contribution to Hyrtanellini contains: a description of a new species from Tajikistan, *Serratella leonidi* Martynov & Palatov, **sp. nov.**; a complementary description and change of generic placement of *Serratella elissa* Jacobus, Zhou & McCafferty, 2009 from Iran, based on material from several sites including the type locality; notes on the morphological affinities of genera *Serratella*, *Torleya* and *Quatica*; phylogenetic reconstruction of Hyrtanellini based on the COI gene that shows a close relationship between the representatives of the three genera mentioned above; and a species list of Western and Central Asia representatives of Hyrtanellini and a key for the immature stage.

## 2. Material and Methods

### 2.1. Sampling, Type Series and Morphological Observation

The present contribution is based on the larval material collected by D. Palatov and J. Bojková in 2016 and 2019, in several regions of Tajikistan and Northern Iran. All specimens were collected by kick sampling using hand nets in different freshwater habitats and preserved in the field in 80–96% ethanol. For long-term preservation, 80–95% ethanol was used. Several specimens of each species discussed in the present contribution were mounted on slides using Canada balsam. Photographs of the specimens mounted on slides were made using a Canon Power Shot A 630 with a Ulab XY-B2T microscope at the National Museum of Natural History, National Academy of Sciences of Ukraine (Kyiv, Ukraine) [further NMNH NASU]; photographs of specimens preserved in alcohol were made using a Leica Z16 APO stereomicroscope, with a Leica DFC450 Digital Camera at the I.I. Schmalhausen Institute of Zoology, National Academy of Sciences of Ukraine. The pictures were subsequently processed with LAS Core 3.8 and the Helicon Focus program. The Scanning Electron Microscopy (SEM) images were made using a Vega3 Tescan. The specimens studied under the scanning electron microscope were first dehydrated using ethanol and dried by critical point drying. Material from the type locality for both species were used for SEM photography.

The type material *Serratella leonidi* **sp. nov.** is deposited in the NMNH NASU [A.V. Martynov’s collection]. The material of *Serratella elissa* used in this contribution is housed in J. Bojková’s collection (Masaryk University, Brno, Czech Republic) [further MUNI] and A.V. Martynov’s collection at the NMNH NASU.

### 2.2. Molecular Study

For main part of the specimens, the total genomic DNA was extracted and mitochondrial cytochrome oxidase subunit I (COI) was sequenced according to Guglya [17]; the procedure was carried out at the Natural History Museum, University of Oslo (Norway). For five specimens, the total DNA was extracted using the Chelex procedure [18] and sequenced according to Trontelj and Utevsky [19] at the V.N. Karazin Kharkiv National University (Ukraine). The GenBank accession numbers for 39 new sequences (8 species from 4 genera) used in the molecular study are given in the Table 1; the nomenclature for the gene sequences follows Chakrabarty et al. [20]. A total of 144 COI sequences of other Hyrtanellini species from 6 genera and three outgroup species (two genera) used in our study were obtained from Cardoni et al. [21], Gattolliat et al. [22], Wakimura et al. [23], Corse et al. [24], Morinière et al. [25], Tenchini et al. [26], Suh et al. [14], Li et al. [15], Xu et al. [16], Behrens-Chapuis et al. [27], Roslin et al. [28] and GenBank (unpublished data).

The alignment of the analyzed sequences were made in BioEdit 7.0.5.3. In studies of species delimitation based on single-locus data, we used both tree- and distance-based methods to make the results more persuasive.

Genetic distances within groups and between taxa were calculated in MEGA 11 [29]. We used IQ-Tree and FigTree v. 1.4.4 for constructing phylogenetic trees from sequence data based on a maximum likelihood (ML) analysis. We used two models of molecular evolution: the Kimura 2-parameter (K2) model [30] and Tamura 3-parameter (T92) model [29], with a gamma distribution (shape parameter = 0.16). This analysis involved 147 nucleotide sequences. The codon positions included were 1st + 2nd + 3rd + Noncoding. All ambiguous positions were removed for each sequence pair (pairwise deletion option). There were 662 positions in the final dataset.

The following taxa were chosen as the outgroup: *Ephemerella mucronata* (Bengtsson, 1909), *Drunella cryptomeria* (Imanishi 1937) and *Drunella lepnevae* (Tshernova 1949).

## 3. Results

### 3.1. Taxonomy

*Serratella leonidi* Martynov & Palatov, **sp. nov.**

**Figure 1:**
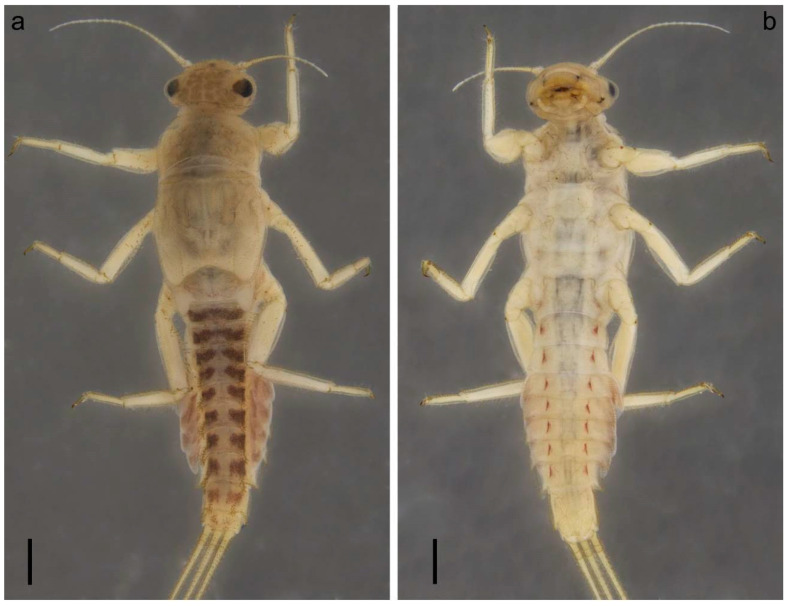
Larva of *Serratella leonidi* Martynov & Palatov, **sp. nov.**, holotype. (**a**) Habitus, dorsal view, (**b**) habitus, ventral view. Scale bar: 0.5 mm.

**Figure 2:**
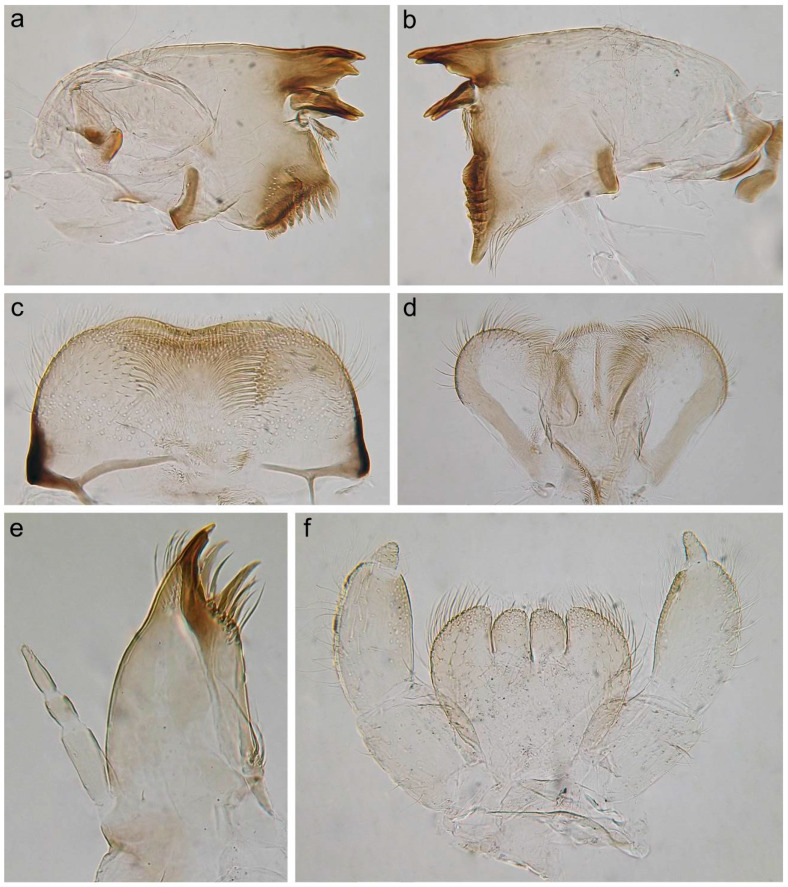
Larva of *Serratella leonidi* Martynov & Palatov, **sp. nov.**, paratype. (**a**) Angulate mandible, (**b**) planate mandible, (**c**) labrum, (**d**) hypopharynx, (**e**) maxilla, (**f**) labium.

**Figure 3:**
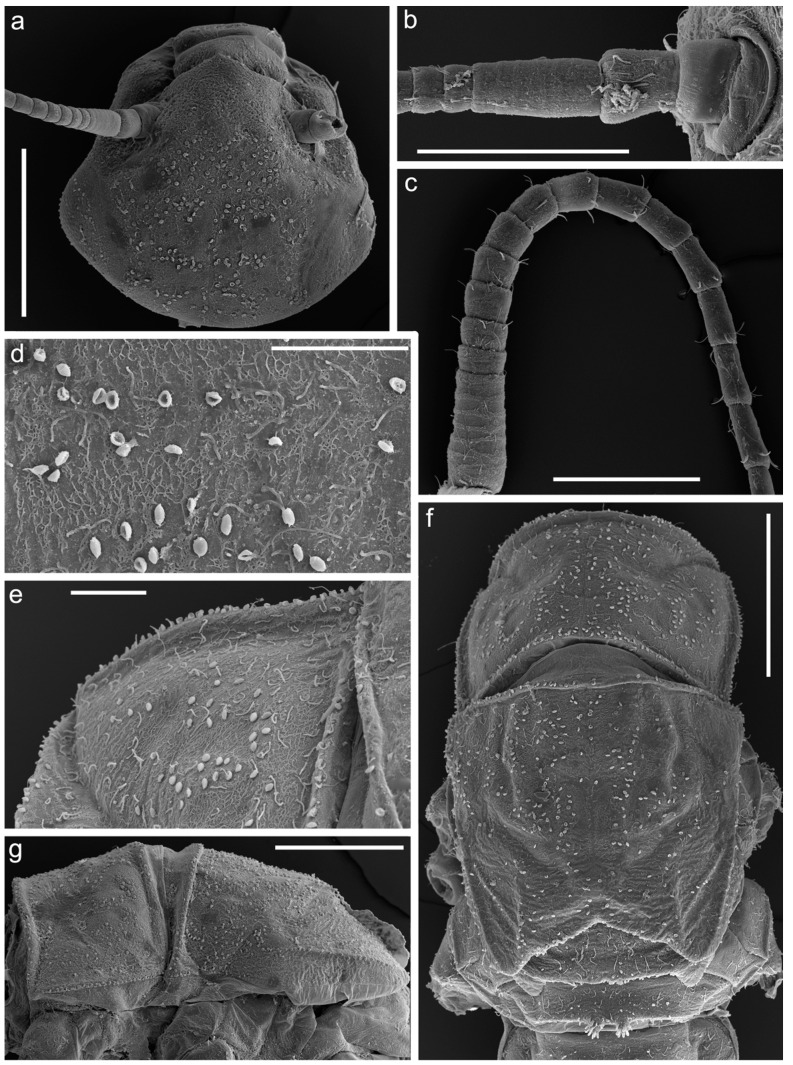
Larva of *Serratella leonidi* Martynov & Palatov, **sp. nov.**, paratypes. (**a**) Head, (**b**) antenna (scapus, pedicelus and part of flagellum), (**c**) flagellum, (**d**,**e**) dorsal surface of pronotum with bubble-like setae, (**f**) thorax, dorsal view, (**g**) thorax, lateral view. Scale bars: (**a**,**g**,**f**)—0.5 mm, (**b**)—0.25 mm, (**c**)—0.2 mm, (**d**,**e**)—0.1 mm.

**Figure 4:**
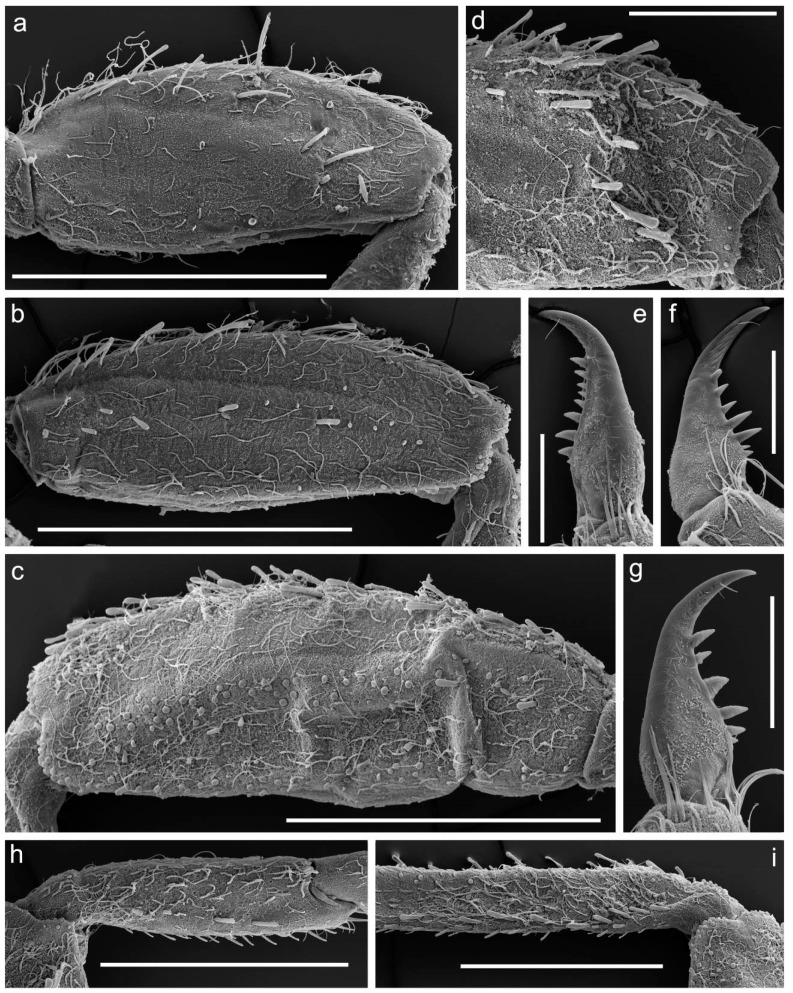
Larva of *Serratella leonidi* Martynov & Palatov, **sp. nov.**, paratypes. (**a**) Forefemur, (**b**) middle femur, (**c**) hind femur, (**d**) transverse row of stout setae on fore femur, (**e**–**g**) tarsal claws, (**h**) fore tibia, (**i**) middle tibia. Scale bars: (**a**–**c**,**h**,**i**)—0.5 mm, (**d**)—0.2 mm, (**e**–**g**)—0.1 mm.

**Figure 5:**
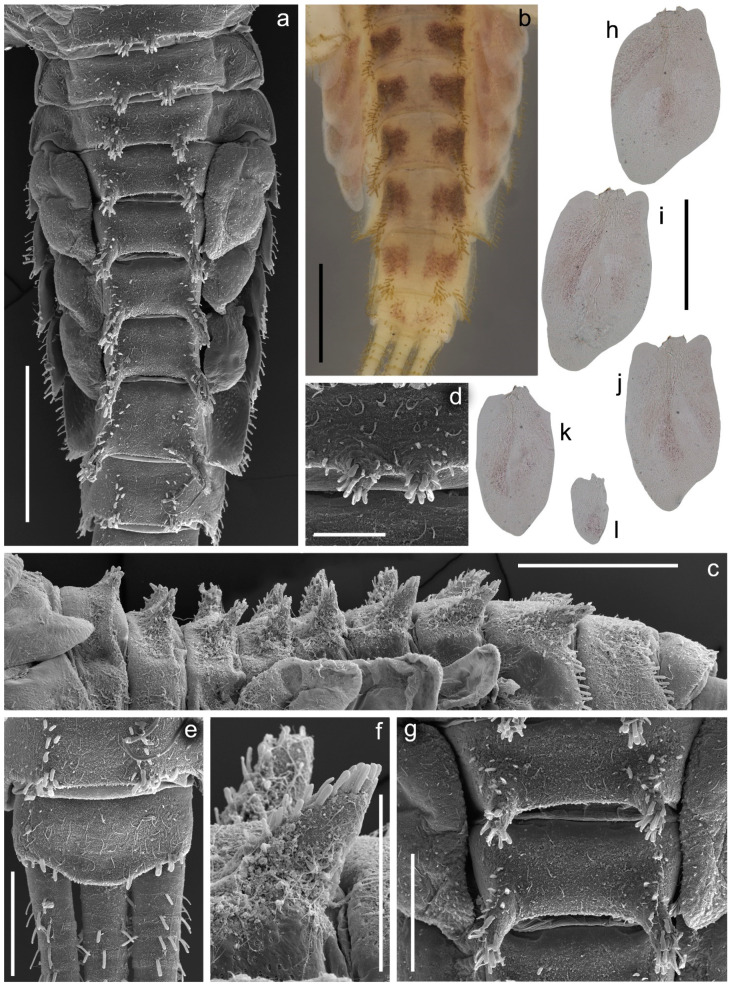
Larva of *Serratella leonidi* Martynov & Palatov, **sp. nov.**, holotype (**b**) and paratypes (**a**,**c**–**l**). (**a**) Abdomen, dorsal view, (**b**) terga V–X, dorsal view, (**c**) abdomen, lateral view, (**d**) submedian tubercles of tergum I, dorsal view, (**e**) terga IX and X, and basal parts of caudal filaments, dorsal view, (**f**) submedian tubercles of tergum VI, dorsal view, (**g**) submedian tubercles of terga IV and V, dorsal view, (**h**–**l**) gills III–VII. Scale bars: (**a**,**b**)—0.5 mm, (**c**)—1 mm; (**d**)—0.1 mm, (**e**–**g**)—0.2 mm; (**h**–**l**)—0.25 mm.

Figure 1, Figure 2, Figure 3, Figure 4 and Figure 5.

**Material examined. *HOLOTYPE***: larva, Tajikistan, Gorno-Badakhshan Autonomous Province, Ishkoshim district, near Kozideh kishlak (village), Pyandzh River, 37.002528, 71.467250, h ~ 2390 m a.s.l., Palatov D.M. leg., 29.VI.2016—*IN Tj6Sersp/1* [NMNH NASU]. ***PARATYPES***: A total of 12 larvae (two larvae mounted on slides # 685, # 686 with Canada balsam), ibid, Palatov D.M. leg., 29.VI.2016—*IN Tj6Sersp/2–7* [NMNH NASU]; 47 larvae, Tajikistan, Dushanbe city, Kofarnihon (Kafirnigan) River opposite the Institute of Zoology and Parasitology of the Academy of Sciences of Tajikistan, 38.524000, 68.836583, h ~ 755 m a.s.l., substrate—large stones, pebbles, rare—sand, Palatov D.M. leg., 20.VI.2016—*IN Tj5Sersp/1–7* [NMNH NASU].

**Etymology**. The species is named in honor of Leonid A. Martynov, the younger son of the first author.

**Diagnosis**. The new species can be easily distinguished from all other representatives of the genus *Serratella* by the combination of characters: (**i**) head and thorax without any tubercles (Figure 3a); (**ii**) well-developed maxillary palp, with distinct articulations (Figure 2e); (**iii**) head, dorsal surface of thorax, middle and hind femora covered with small bubble-like setae (Figure 3a,d–f and Figure 4b,c); (**iv**) forefemur with irregular, discontinuous, transverse row of long stout setae, rounded apically, with slightly divergent margins (Figure 4a); (**v**) tarsal claw with 5–9 sharp denticles, and with one to several subapical setae (Figure 4e–g); (**vi**) terga I–IX with paired distinct projections posteriorly; those on tergum IX are the smallest compared to those on the other terga (Figure 5a,c,e,g); (**vii**) paired abdominal projections covered with long stout setae, rounded apically, with slightly divergent or subparallel margins (Figure 5a–g); and (**viii**) posterior margins of terga VIII–IX with rows of above-mentioned type of stout setae, runs from paired projections to lateral margins of segment; posterior margin of tergum X also has such stout setae, but they are shorter than on previous segments (Figure 5c,e).

**Description**. *Larva:* Body length 5.0–5.8 mm; caudal filaments 3.2–4.1 mm. Body, legs and caudal filaments are yellow; abdominal terga I–IX with median broad brown band; coloration of terga III–IX uniformly colored yellow, with brown transverse elongated spots not connected with each other; terga II and III with transverse brown band; tergum X with a pair of indistinct submedian smudges (Figure 1a and Figure 5b). Sterna II–VIII with a pair of longitudinal hatches, indistinct on sterna I and IX (Figure 1a,b and Figure 5b). Paired abdominal process brown only at the base.

***Head***: Genae moderately developed, rounded, without any tubercles or ridges. Surface of head covered with scattered hair-like setae (main number on genae), and small bubble-like setae (main number on vertex) (Figure 3a).

*Mouthparts*: Labrum wide, anterolateral angles rounded; anteromedian emargination wide and shallow (Figure 2c). Dorsal surface of labrum with paired groups of long, thin, hair-like setae on lateral lobes; anterolateral angles and adjacent part of dorsal surface covered with long, stout, hair-like setae with smooth and feathered margins; anterior margin of labrum; dorsal surface along the labrum margin covered with numerous short, feathered setae and its anterior margin with six very short, stout setae feathered apically. Additionally, dorsal surface of labrum with densely scattered short, thin, hair-like setae and scale sockets. On both mandibles outer incisor with three teeth; inner incisor with two teeth (Figure 2a,b). Central part of outer margin of mandibles and adjacent area of dorsal surface with a group of long, thin, hair-like setae. Dorsal surface of mandibles also covered with scale sockets. Right mandible with a row of eight long, stout, hair-like setae, feathered marginally. Superlinguae of hypopharynx with rounded apices covered with mainly long, stout, hair-like setae (Figure 2d). Outer margins of superlinguae without setae. Lingua densely covered with short, thin, hair-like setae. Rows of setae on lateral sides of lingua consist of 6–7 elongated, pointed, stout setae. Maxillary palp elongated, three-segmented, with distinct articulation (Figure 2e); segments I and II distinctly longer than segment III; distal segment III with margins convergent from middle, rounded at the tip, its inner margin covered with fine setae apically and distally. Galea-lacinia with a row of 6–7 long, pointed, stout setae with feathered margins, and two dentisetae on inner margin; a group of about 10 long setae near of bases of maxillary canines; one robust denticle rounded apically on inner margin of galea-lacinia above dentisetae. Base of galea-lacinia with a group of 5–6 long, hair-like, stout setae with feathered margins on inner margin; one elongated stout seta near base of maxillary palp. Dorsal surface of mentum with a group of 6–7 long, thin, hair-like setae near base of glossae (Figure 2f). Ventral surface of mentum with densely scattered scale sockets. Dorsal and ventral surfaces of glossae and paraglossae tips covered with differently sized stout, hair-like setae; the same type of setae covers ventral surface of paraglossae along outer margin. Labial palp three-segmented; segments I and II subequal in length, flattened; segment III short, rounded apically, covered with numerous fine setae, with convergent margins; segment III length/width ratio at base: 1.3–1.5; segments I and II outer and inner margins (mainly in distal half of segment II) covered with numerous thin, hair-like setae; segments I and II outer margins and areas along of ventral surface covered with long, stout, hair-like setae and spine-like setae.

***Thorax***: Ridges and tubercles absent on thoracic surface. Anterolateral angels of pronotum without any distinct projections. Mesothorax without anterolateral projections. Dorsal surface of thorax covered with scattered short, hair-like setae and small bubble-like setae (Figure 3d–g).

Femora of all legs slightly flattened; length/width ratio of forefemur 2.1–2.2; middle femur 2.3–2.4; hind femur 2.7–2.9; no ridges on all femora. Average length ratio of legs parts (femur:tibia:tarsus): foreleg 1.68:1.54:1.00; middle leg 1.68:1.72:1.00; and hind leg 1.79:2.00:1.00. Outer margins of all femora without apical projections.

Dorsal surface of forefemur covered with scattered long, thin, hair-like setae (occasionally in groups of 2–3 setae), several small bubble-like setae and area with spine-like microtrichia near basal edge; basal part of femoral surface along outer margin with several long, mainly rounded apically, stout setae (occasionally with slightly divergent margins); distal half of femoral surface with irregular, discontinuous, transverse band of long, rounded apically, stout setae with slightly divergent margins. Outer margin of forefemur with irregular row of the same type of stout setae and a few long, thin, hair-like setae (Figure 4a,d). Outer margin of foretibia and tarsus with a few long, thin, hair-like setae only (often grouped into 2–3 setae); their ventral surfaces with scattered mainly long, thin, hair-like setae, almost grouped into 2–4 setae; the setae most numerous on tarsus and distal margin of tibia. Dorsal surface of foretibia with longitudinal row of 4–6 long, rounded apically, stout setae with slightly divergent margins and a few thin, mainly long, hair-like setae (Figure 4h). Inner margin of foretibia, its adjacent area of ventral surface, and inner margin of tarsus with a row of long, rounded, stout setae and a few thin, hair-like setae.

Dorsal surface of middle and hind femora with scattered, mainly long, thin, hair-like setae and small bubble-like setae; basal half with area of spine-like microtrichia; central part of surface with several long, apically rounded, stout setae with slightly divergent margins (4–5 setae on middle femur; 1–2 on hind femur) (Figure 4b,c). Outer margins of femora and adjacent areas of dorsal surfaces covered with above-mentioned type of stout setae and scattered long, thin, hair-like setae. Dorsal surfaces of middle and hind tibiae with irregular, discontinuous row of the same type of 7–17 stout setae, runs along patella–tibial suture and inner margin; scattered long, thin, hair-like setae also covered the dorsal surfaces. Inner margins of middle and hind tibiae, and its adjacent area on ventral surface with a row of mainly long, stout setae. Outer margins of the middle tibia with several stout setae; hind tibia with 10–15 stout setae (Figure 4i); additionally, hind tibia margins with a few thin, hair-like setae (often grouped in pairs). Outer margins, dorsal and ventral surfaces of middle and hind tarsi with thin, mainly long, hair-like setae (mainly in groups by two setae); inner margins of tarsi with a row of long stout setae.

Tarsal claw of all legs hooked, with 5–9 sharp denticles, and one to several subapical setae (Figure 4e–g).

***Abdomen***: Terga I–IX with paired distinct projections; those on terga IV–VIII strongest; those on tergum IX smallest among those on all other terga. All the projections and adjacent areas of terga covered with mainly elongated, apically rounded, stout setae with slightly divergent or subparallel margins; distant from projections stout setae distinctly shorter and tiny (Figure 5a–g). All terga covered with spine-like microtrichia and few thin hair-like setae; the microtrichia most numerous and distinct in medial areas of terga; in addition, few scattered small bubble-like setae covered terga. Lateral areas of terga IV–VIII additionally densely covered with round scale sockets. Segments IV–IX with posterolateral projections. Lateral margins of segments IV–IX covered with mainly long, apically rounded, stout setae with slightly divergent margins (Figure 5a). Posterior margins of terga I–VII with scattered thin, hair-like setae and spine-like microtrichia only; posterior margins of terga VIII–IX additionally with rows of above-mentioned kind of stout setae runs from paired projections to lateral margins (Figure 5c); posterior margin of terga X also with stout setae, but they are shorter than on previous segments (Figure 5e).

Caudal filaments subequal in length; posterior area of their segments bears discontinuous rings of long, rounded, unicolored apically stout setae, with subparallel or slightly divergent margins.

Winged stages: unknown.

**Distribution and biology.** The new species is probably endemic to Central Asia. Based on current knowledge, it is distributed locally, in the reaches of the upper Amu Darya basin. *Serratella leonidi* **sp. nov.** inhabits the largest mountain rivers, e.g., the Kofarnihon River in central Tajikistan and the Pyanzh River as the natural border between Tajikistan and Afghanistan. The larvae of the new species inhabit sections with rocky substrates (Figure 11a,b), and were found within the river sections with pebbles, stones and roots, with current velocity about 0.3–0.7 m/s. Obviously, the size of the watercourse, the speed of the flow, and the nature of the bottom are key factors for this species distribution. Thus, *Serratella leonidi* **sp. nov.** was found both in a high mountain river with an average summer temperature of 10–13 °C, and in a foothill river that warms up to 20 °C or more in the summer. There is a high probability that *Serratella leonidi* **sp. nov.** will be found in other rivers with similar characteristics, such as the Vaksh, Vakhsu, Kunduz, Bartang and Surkhan. This species is not expected in lowland watercourses; we failed to find it during a special study of the lower flow of the Vaksh River where fine sand becomes the predominant substrate. Thus, the species can be found with high probability in the northern part of Afghanistan, south-eastern part of Uzbekistan and almost throughout Tajikistan.

***Torleya elissa* (Jacobus, Zhou & McCafferty, 2009) comb. nov.**

*Serratella elissa* Jacobus, Zhou & McCafferty, 2009

**Figure 6:**
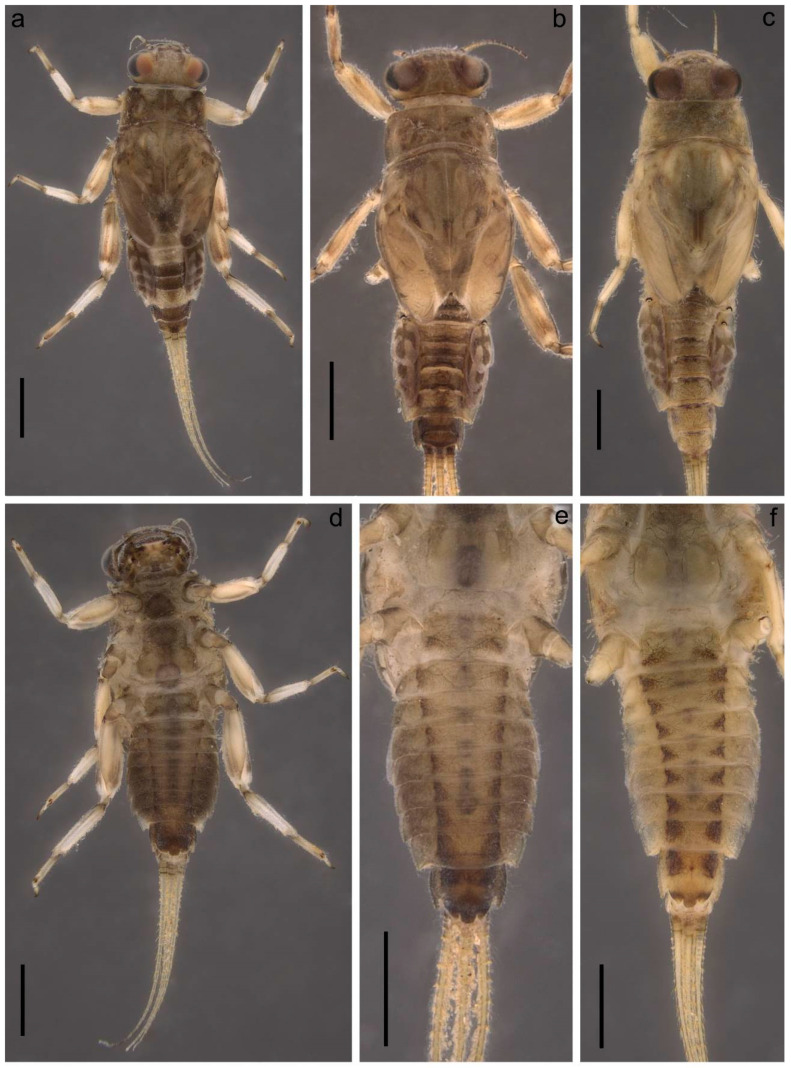
Coloration of larva of *Torleya elissa* (Jacobus, Zhou & McCafferty, 2009). (**a**–**c**) Habitus, dorsal view, (**d**) habitus, ventral view, (**e**,**f**) abdomen, ventral view. Scale bar: 1 mm.

**Figure 7:**
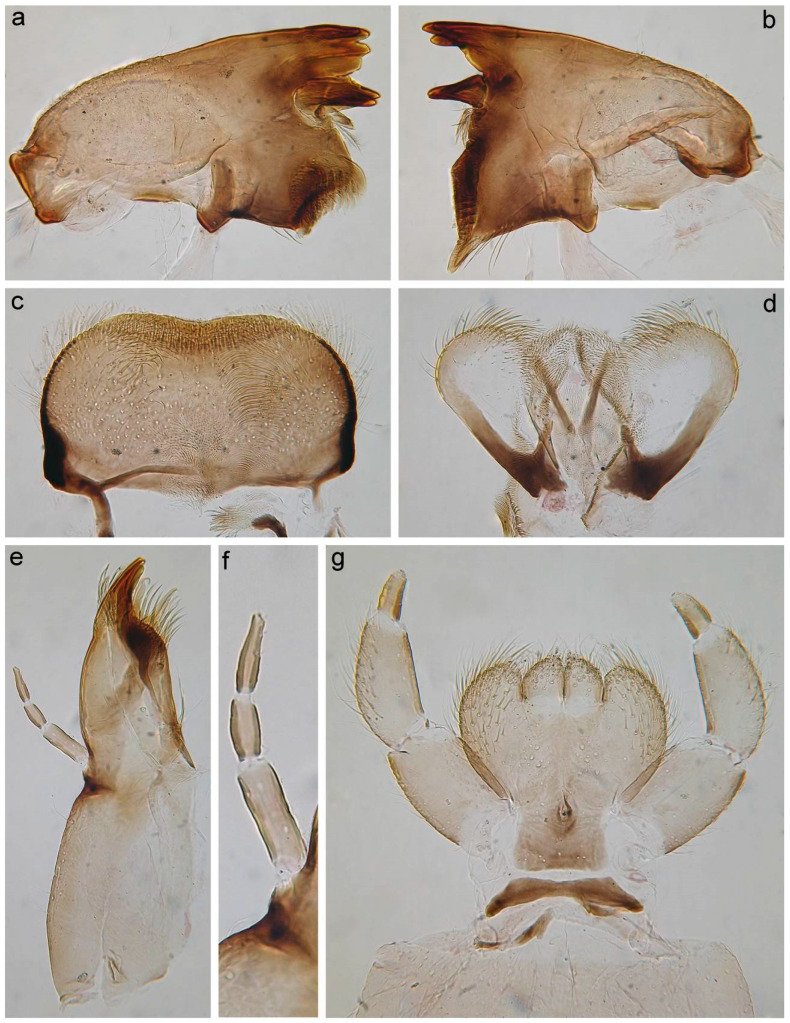
Larva of *Torleya elissa* (Jacobus, Zhou & McCafferty, 2009). (**a**) Angulate mandible, (**b**) planate mandible, (**c**) labrum, (**d**) hypopharynx, (**e**) maxilla, (**f**) maxillary palp, (**g**) labium.

**Figure 8:**
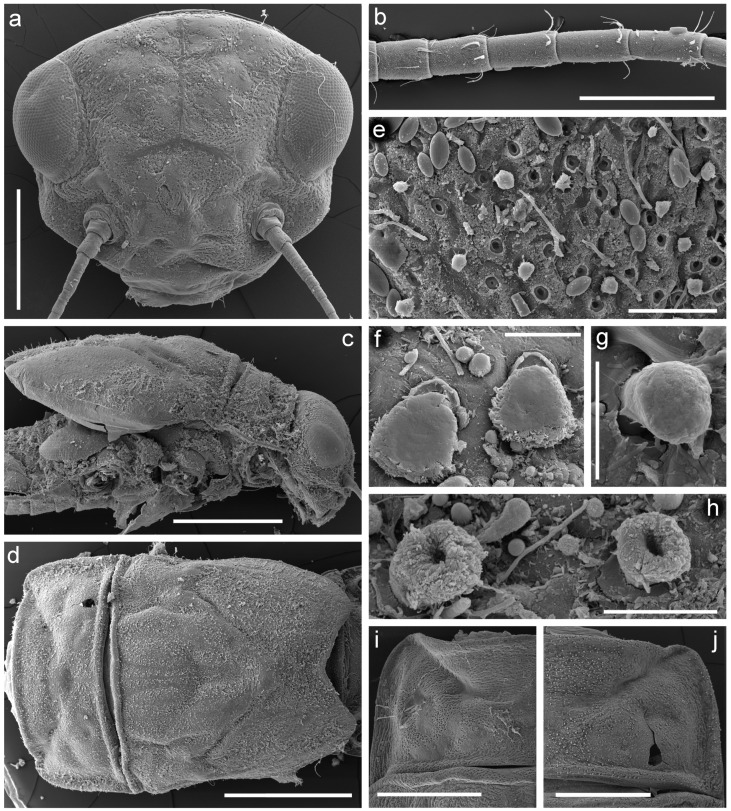
Larva of *Torleya elissa* (Jacobus, Zhou & McCafferty, 2009). (**a**) Head; (**b**) flagellum, (**c**) thorax, lateral view, (**d**) thorax, dorsal view, (**e**) setae on dorsal surface of pronotum, (**f**–**h**) setae of pronotum, (**i**) pronotum with a few setae, (**j**) pronotum with numerous setae. Scale bars: (**a**,**i**,**j**)—0.5 mm, (**b**)—0.2 mm, (**c**,**d**)—1 mm, (**e**)—0.05 mm, (**f**–**h**)—0.01 mm.

**Figure 9:**
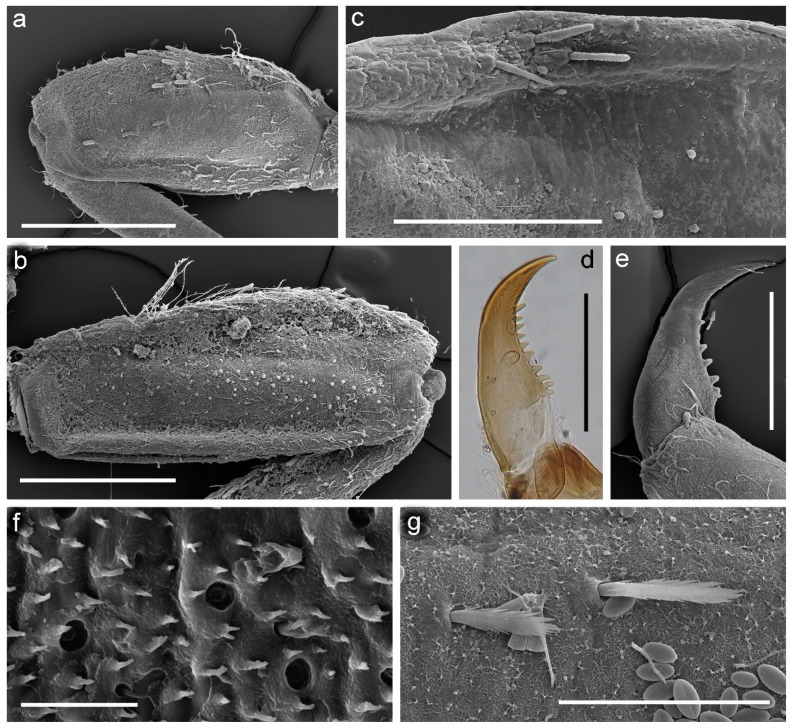
Larva of *Torleya elissa* (Jacobus, Zhou & McCafferty, 2009). (**a**) fore femur, (**b**) middle femur, (**c**) group of stout setae on fore femur, (**d**,**e**) tarsal claws, (**f**) dorsal surface of femur, (**g**) setae on dorsal surface of hind tibia. Scale bars: (**a**,**b**)—0.5 mm, (**c**)—0.25 mm, (**d**,**e**)—0.2 mm, (**f**)—0.02 mm, (**g**)—0.1 mm.

**Figure 10:**
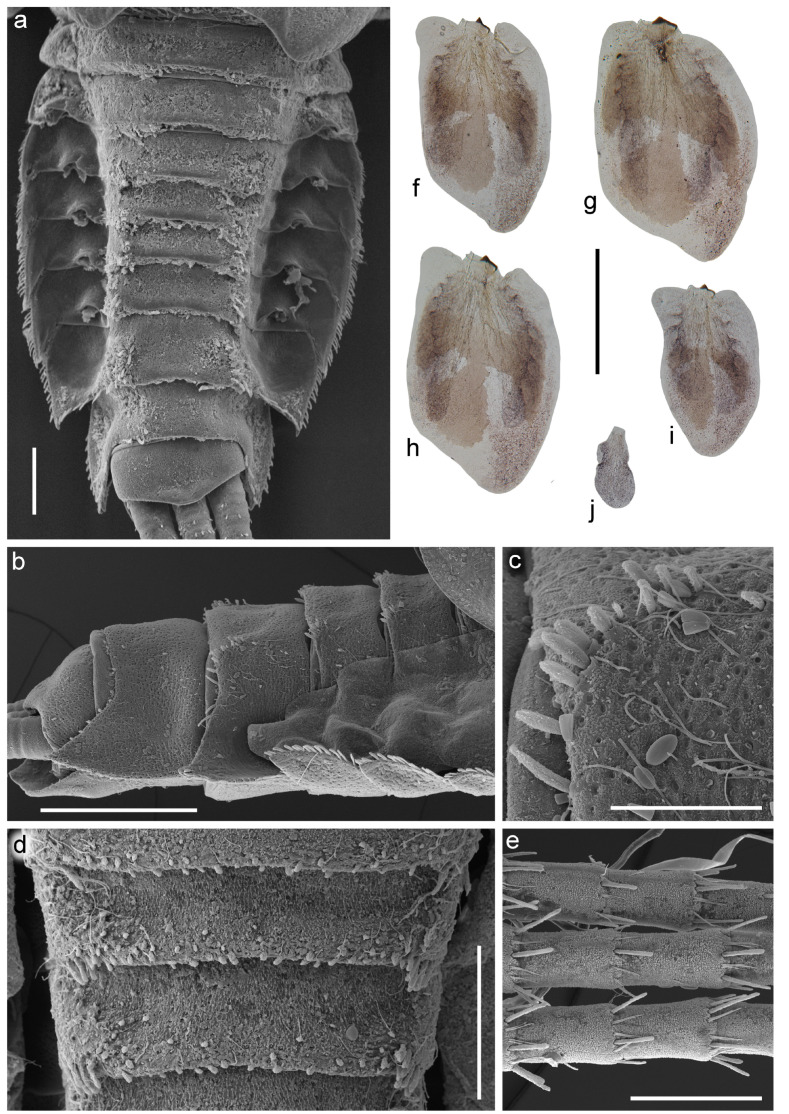
Larva of *Torleya elissa* (Jacobus, Zhou & McCafferty, 2009). (**a**) Abdomen, dorsal view, (**b**) terga VI–X, lateral view, (**c**) setae of tubercles on tergum VIII, (**d**) posterior margins of terga IV–VI, (**e**) caudal filaments, dorsal view, (**f**–**j**) gills III–VII. Scale bars: (**a**)—0.3 mm, (**b**,**f**–**j**)—0.5 mm, (**c**)—0.1 mm, (**d**)—0.25 mm, (**e**)—0.2 mm.

Figure 6, Figure 7, Figure 8, Figure 9 and Figure 10

The distinguishing characteristics of this species were provided in a brief but informative original description, which supported the figures of the general dorsal view of larva, maxilla and tarsal claw [6]. Herein is a complementary description of some characteristics including variable ones and illustrations useful for the species determination (Figure 6, Figure 7, Figure 8, Figure 9 and Figure 10). All additional data are based on material collected in northern Iran in several areas, including the type locality of *T. elissa*.

**Material examined.** A total of 28 larvae, Iran, Lorestan Province, Dare Daei River—left tributaries of the Tenge-Gendumer-Darre-Luku River, 33.186067, 49.510117, h—2244 m a.s.l., 13.VI.2019, Palatov D.M. leg.—*IN Iran9Sersp1/1–4* [NMNH NASU]; one larva, Iran, Lorestan Province, Tenge-Kholeven River, upstream of the Cham Chit village, 33.379546, 48.973726, h—1338 m a.s.l., 29.VI.2019, Palatov D.M. leg.—*IN Iran8Sersp1/1* [NMNH NASU]; 15 larvae (slides # 669, # 691), Iran, Gilan Province, S of Siahkal city, Machian, left tributary of Bala Rud River, Lunak waterfalls, 37.008611, 49.864167, 16.V.2016, Bojková J., Soldán T., Imanpour Namin J. leg.—*type locality*—MUNI (10 larvae), NMNH NASU (*IN Iran6Serel/1–2*—5 larvae, including two slides); 6 larvae, Iran, Gilan Province, south of Paein mahale Khara Rud (S of Pashaki village), unnamed brook, right fork of Khara Rud River, 37.038889, 49.790833, 12.V.2016, Bojková J., Soldán T., Imanpour Namin J. leg. [MUNI]; 66 larvae, Iran, Gilan Province, south of Tushi village (S of Siahkal city), Bala Rud Shamrood, right tributary of Sefid Rud River, 37.049977, 49.898749, 16.V.2016, Bojková J., Soldán T., Imanpour Namin J. leg.—MUNI (59 larvae), NMNH NASU (*IN Iran7Serel/1*—9 larvae); 1 larva, Iran, Ardabil Province, Hakim Geshlaghi chayi, left tributary of Hakim Geshlaghi, in Nir town, 38.032222, 47.996667, 18.V.2016, Bojková J., Soldán T., Imanpour Namin J. leg. [MUNI]; 73 larvae, one larval exuvium (slide # 671), Iran, Gilan Province, above Chelvand village (S of Lavandvil town), Chelavand River, ca 2.5 km from its inflow to the Caspian Sea, 38.288889, 48.859722, 19.V.2016, Bojková J., Soldán T., Imanpour Namin J. leg.—MUNI (58 larvae), NMNH NASU (*IN Iran5Serel/1–2*—15 larvae, one larval exuvium mounted on slide); 167 larvae (slide # 670, 692), Iran, Chaharmahal and Bakhtiari Province, left tributary of Zayandeh Rud, NE of Dimeh, 32.519300, 50.227333, h –2215 m a.s.l., 01.V.2016, Bojková J., Soldán T., Imanpour Namin J. leg.—MUNI (149 larvae), NMNH NASU (*IN Iran4Serel/1–5*—18 larvae, including two slides).

**Complementary description.** Head, especially vertex, bears tiny, apically rounded setae, and setae with divergent margins; both types of setae with plumose tips and sometimes margins (on SEM photos they look as clavate setae) (Figure 8a). Flagellar segments with a groups of two–three hair-like setae near posterior margin (Figure 8b). Segment III of labial palp short of elongated (Figure 7g); as a result, length/width ratio at base significantly vary 1.2–2.0 (average 1.6).

Dorsal surface of thorax covered with tiny, apically rounded setae, and setae with divergent margins; the largest number of the setae situated on forewing pads and central part of mesothorax (Figure 8c–h,j); often rounded setae almost absent (Figure 8i); thorax also covered with thin, hair-like setae.

Dorsal surface of thorax, legs and abdomen covered with rounded scale sockets (Figure 8e). Thorax without distinct ridges and protuberances; only blunt posterior projection between forewing pads (MP) visible in lateral view (Figure 8c). Denticles of tarsal claw bluntly pointed, their number varies from six to eleven (Figure 9d,e).

All femora slightly flattened, without apical projections on outer margins; forefemur length/width ratio is 1.9–2.4 (average 2.1); middle femur 1.9–2.6 (average 2.3); and hind femur 2.1–2.7 (average 2.4). Average length ratio of leg segments (femur:tibia:tarsus): foreleg 1.54:1.30:1.00; middle leg 1.69:1.45:1.00; and hind leg 1.78:1.62:1.00.

Abdominal terga from dirty yellow to light brown; in some dark specimens, terga IV, V and VIII distinctly lighter (Figure 6a–c). Sterna I–VIII with paired dark submedian smudges; sometimes merged smudges looks like two longitudinal lines. Sternum IX distinctly darker than all other sterna (Figure 6d–f). Occasionally, several stout setae on femora, tibiae and posterior margins of terga are distinctly black.

Segments of the central part of caudal filaments fringed with long, mainly pointed, bluntly pointed or rounded stout setae (Figure 10e); some of setae stout, hair-like, longer than corresponding segment. Several stout setae in basal part of filaments short and apically rounded.

**Figure 11:**
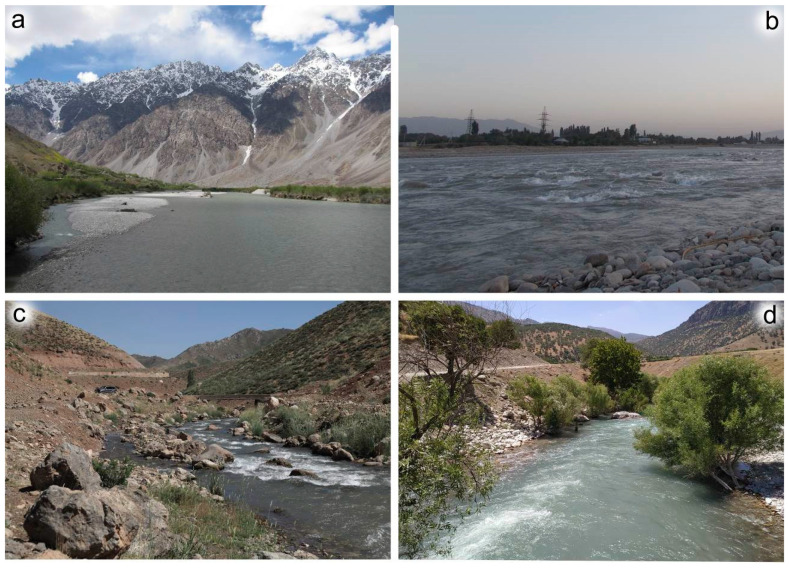
Habitats of *Serratella leonidi* Martynov & Palatov, **sp. nov.** (**a**,**b**) and *Torleya elissa* (Jacobus, Zhou & McCafferty, 2009) (**c**,**d**): (**a**) Pyandzh River (Tajikistan, June 2016), (**b**) Kofarnihon (=Kafirnigan) River (Tajikistan, June 2016), (**c**) nameless river—left tributaries of the Tenge-Gendumer-Darre-Luku River (Iran, June 2019), (**d**) Tenge-Kholeven River (Iran, June 2019).

**Figure 12:**
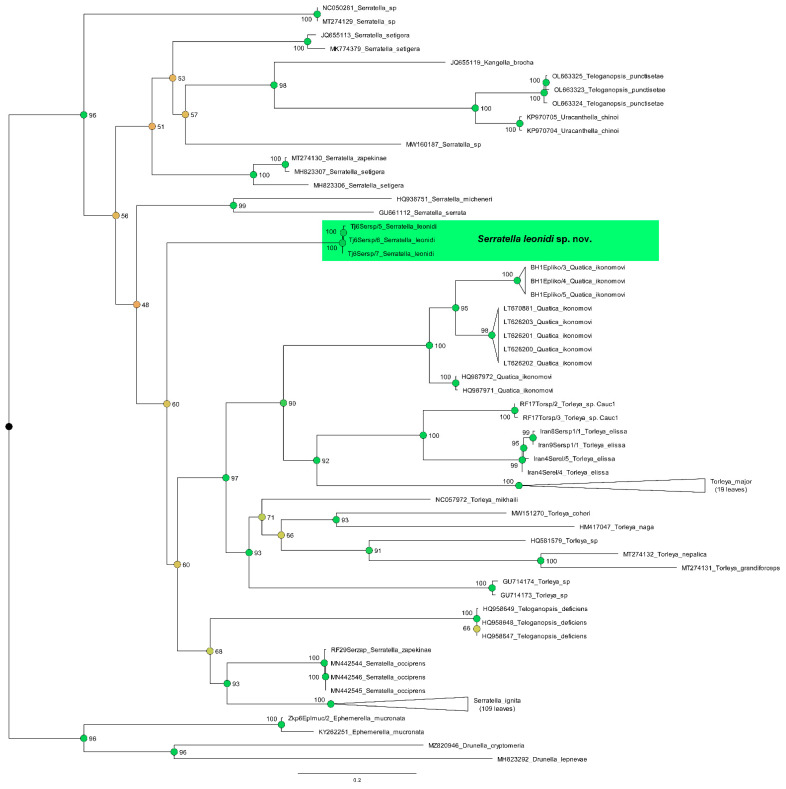
Maximum likelihood tree including representatives of tribe Hyrtanellini. Branches provided with Bootstrap supports (BS). Support values are colored based on confidence at internal nodes; from yellow (low) to green (high). Green background marks new species.

**Figure 13:**
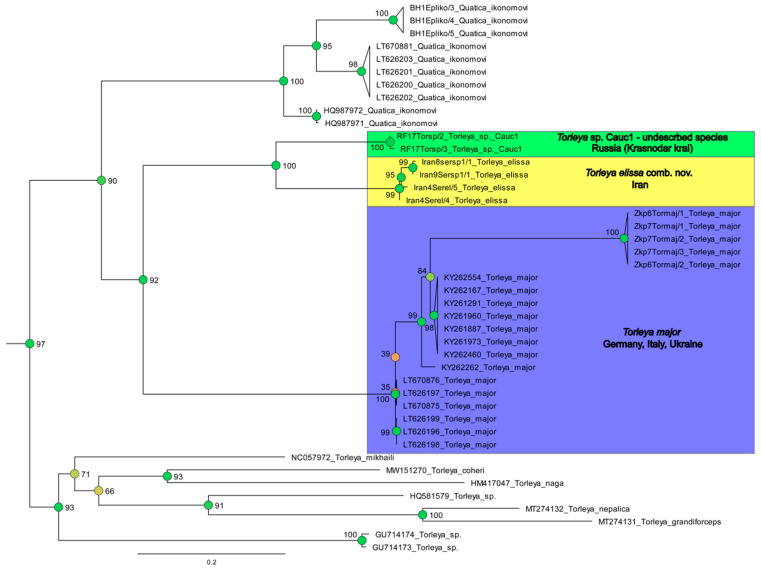
Part of Maximum likelihood tree on tribe Hyrtanellini showing lineage with *Torleya* Lestage, 1917 and *Quatica* Jacobus & McCafferty, 2008. Branches provided with Bootstrap supports (BS). Support values are colored based on confidence at internal nodes; from yellow (low) to green (high). Green, yellow and purple backgrounds mark different species.

**Remark**. Jacobus et al. [6] in the contribution with original description of *S. elissa* mentioned that they only provisionally consider this species to be part of the genus *Serratella* Edmunds, 1959. On our opinion, despite the absence of distinct borders in all stages, there are characteristics such as the absence of paired abdominal projections, which approximate *S. elissa* to the genus *Torleya* Lestage, 1917. Additionally, as showed on our ML tree (Figure 11, Figure 12 and Figure 13), the relation of *S. elissa* with other sequenced Hyrtanellini are the reasons for its placing within *Torleya*.

### 3.2. Molecular Results

In this study, we used a series of COI sequences of Hyrtanellini species which were not in doubt. Based on position of some Operational Taxonomic Units’ sequences (OTUs’ sequences), vouchers that were not determined up to species level on our ML tree were assumed, in some cases, to represent new taxa that have not yet been described.

Our phylogenetic reconstruction based on the COI gene showed the absence of a distinct division between *Serratella* and *Torleya* (Figure 12). Simultaneously, all *Torleya* OTUs analyzed by us were placed together (Figure 13). This ML tree also clearly confirmed the suggestion of Jacobus et al. [6], that the genus *Serratella* is not a monophyletic taxon. The genus representatives did not place within one clade on the ML tree and were often significantly distant from each other (Figure 12). This contradicts the results obtained by Ogden et al. [31] and Xu et al. [16] based on detailed studies of molecular and morphological data, but using only a few species belonging to two genera.

The position of the type species of the genus *Quatica* (i.e., *Q. ikonomovi* (Puthz, 1971)) is defined on the ML tree in one clade with representatives of *Torleya* (Figure 12 and Figure 13). Together with the absence of a significant division of these genera, and morphological notes on *Quatica* mentioned below, should initiate a further detail morphological and molecular study of this genus’ representatives. We cannot exclude that there is a high possibility that *Quatica* will be considered a junior synonym of *Torleya* (Figure 12 and Figure 13).

*Serratella leonidi* Martynov & Palatov, **sp. nov.** forms a significantly diverged lineage on the ML tree (Figure 12). The genetic distances between this new species and other sequenced Hyrtanellini which occur within Western and Central Asia are large (K2—0.207–0.232; T92+G—0.675–0.836; Table 2). The genetic distance within the species is 0.003 (K2/T92+G, n = 3); this small value can be partially caused by the collection of all the specimens in one locality.

Based on the ML tree, *T. elissa* is closely related to the *Torleya* representatives (Figure 12 and Figure 13), and it is partially reasoned change of the generic position of this species. The genetic distances within the species are 0.013/0.014 (K2/T92+G, n = 4).

**Figure 14:**
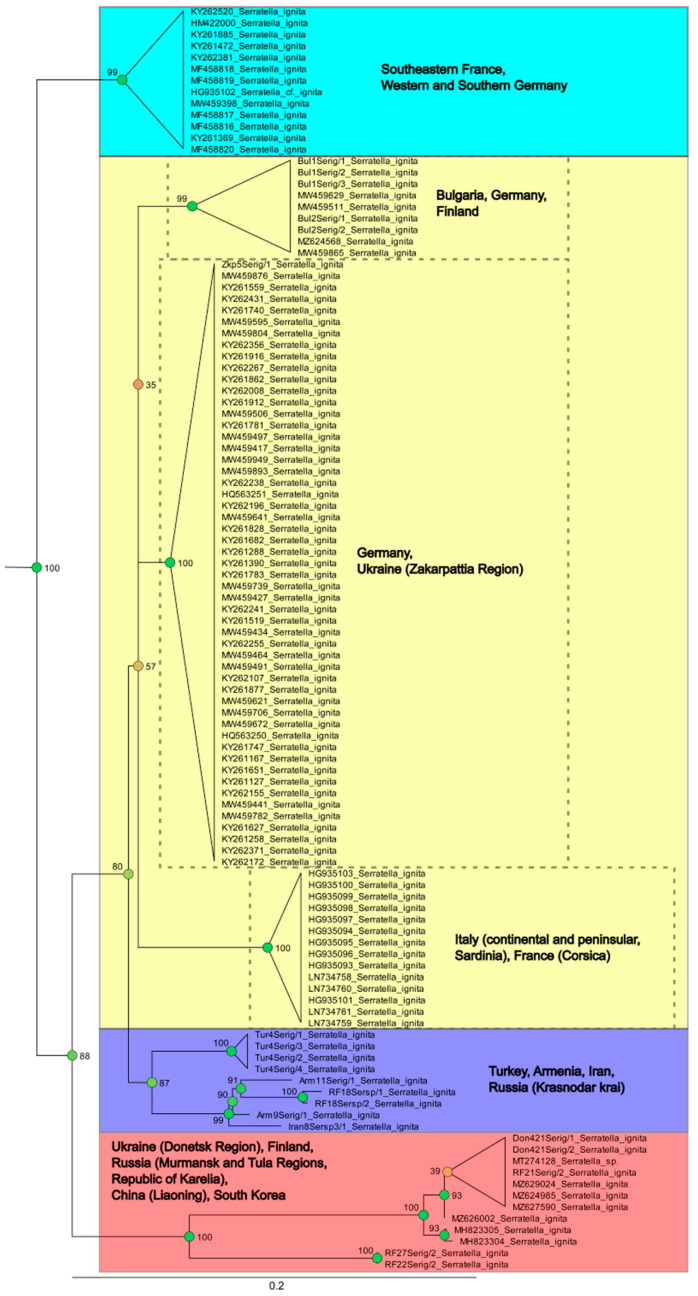
Part of Maximum likelihood tree on tribe Hyrtanellini showing lineage with *Serratella ignita* (Poda, 1761). Branches provided with Bootstrap supports (BS). Support values are colored based on confidence at internal nodes; from yellow (low) to green (high). Blue, yellow, purple and coral backgrounds mark the most distinct clades within lineage with *S. ignita*.

Gattoliat et al. [22] registered a single haplotype (from southern France) of *S. ignita* which significantly diverged from Corsican specimens (main territory of their study). They mentioned that the genus is supposed to be monospecific in Europe and no morphological differences were observed, suggesting continental cryptic species in *Serratella*. On our ML tree, *S. ignita* (n = 109) was also presented by distinct two lineages (Figure 14) and support the presence of cryptic species within southeastern France, and South and West Germany. In addition, the clade of samples from Western Asia (Turkey, Iran, Armenia and Russian Krasnodar krai) and the clade of samples from Ukraine (Donetsk Region), Finland, Russia (Murmansk and Tula Regions, Republic of Karelia), China (Liaoning) and South Korea, were distinct but not significantly diverged. Notably, samples from the Donetsk Region (Ukraine) where the species presented with a geographically isolated population and considered a glacial relict [32], are the most closely relate to samples from Finland and Russia (Tula and Murmansk Regions, Republic of Karelia), in contrast to samples from the Zakarpattia Region (Ukraine) that are situated in another clade with specimens from Germany. The presence of the clade of samples that belong to the territories of China (Liaoning) and South Korea cannot be currently explained by zoogeographical consideration. Another large clade joined samples from Bulgaria, Germany, Finland, Ukraine (Zakarpattia Region), Italy (continental and peninsular, Sardinia), France (Corsica); they are divided into three smaller groups, but their distance and bootstrap support are low. Generally, in our opinion, it is doubtful whether the presence of the mayfly species with such a large distribution that *S. ignita* has, and the series of cryptic species will be described from different parts of Eurasia.

Within the north-eastern Black Sea coastal area (Krasnodar krai of Russia), we found larvae of *Torleya* species (*Torleya* sp. Cauc1) which are morphologically and genetically closely related to *T. elissa* and *Torleya major* (Klapálek, 1905). The genetic distance (K2/T92+G) between this OTU and *T. elissa* (0.148/0.349) and *T. major* (0.216/0.755) are large (Figure 12 and Figure 13, Table 2). Currently, there are not enough data for morphological description of the *Torleya* sp. Cauc1 as a new species, but this information will be useful for the study of Caucasian freshwaters diversity. The registered Caucasian taxon, as well as *Centroptilum volodymyri* Martynov, Godunko & Palatov, 2022 that showed large genetic distances between Caucasian and Iranian populations (K2—0.13) which can be a separate cryptic species, are new evidences that will help to understand the origin and relationship of Caucasian and Iranian mayfly faunas [33].

## 4. Discussion

### 4.1. List of Species, Currently Known Distribution and Key for Larvae of Hyrtanellini Allen, 1980 of Western and Central Asia

The composition and boundaries of *Serratella*, *Torleya* and *Quatica* are debatable [2,16,34,35,36]; the generic position of some of their representatives will be changed in the future, but herein (see in species list, key, etc.) they are provided according to the current systematic position.

For now, eleven species of the tribe Hyrtanellini are known from Western and Central Asia (see also Table 3):

*Serratella* Edmunds, 1959

*Serratella ignita* (Poda, 1761)

*Serratella leonidi* Martynov & Palatov, **sp. nov.**

*Serratella karia* (Kazanci, 1990)

*Torleya* Lestage, 1917

*Torleya elissa* (Jacobus, Zhou & McCafferty, 2009) **comb. nov.**

*Torleya major* (Klapálek, 1905)

*Quatica* Jacobus & McCafferty, 2008

*Quatica euphratica* (Kazanci, 1987)

*Quatica ikonomovi* (Puthz, 1971)

*Teloganopsis* Ulmer, 1939

*Teloganopsis bauernfeindi* (Thomas, Marie & Dia, 1999 [in Marie, Dia and Thomas])

*Teloganopsis maculocaudata* (Ikonomov, 1961)

*Teloganopsis mesoleuca* (Brauer, 1857)

*Teloganopsis subsolana* (Allen, 1973)

Furthermore, the one species mentioned above as the undescribed *Torleya* sp. Cauc1, can be found in some West Asian countries.

The state of our knowledge of the specific differences the adults and larvae of Hyrtanellini is not the same. The imaginal stage is only described for three species from target territories, namely in *S. ignita*, *T. major* and *Q. euphratica*. However, all twelve species are known at larval stage. Thus, there is no useful key for adults of Hyrtanellini, but a determination key for larvae is necessary and it is provided below. Part of the key on *Teloganopsis* is based on Marie et al. [11]:

**1** Claw with one median row of denticles, where preapical denticle stout and larger than all other species—**2** (genus *Teloganopsis* Ulmer, 1939).

– Claw with one median row of denticles, where preapical denticle not stouter and not larger than all other—**5**.

**2(1)** Antennae shorter or subequal in length to fore tibia—***Teloganopsis subsolana* (Allen**, **1973)**.

– Antennae at least two times longer than fore tibia length—**3**.

**3(2)** Apex of maxilla is wide, spoon-like, with concavity; head, pronotum and mesonotum with distinct, narrow median line as wide as median suture; outer margin and dorsal surface of femora with short and widely rounded setae—***Teloganopsis bauernfeindi*** (**Thomas**, **Marie and Dia**, **1999 [in Marie**, **Dia and Thomas]**).

– Apex of maxilla narrow; head, pronotum and mesonotum with whitish median line distinctly wider than median suture; outer margin and dorsal surface of femora with long and narrow setae—**4**.

**4(3)** Third segment of labial palp is narrow and rounded, approximately three times longer than width basally; cerci with dark brown band 2/5 from base or situated medially—***Teloganopsis mesoleuca* (Brauer**, **1857)**.

– Third segment of labial palp relatively triangular, approximately two times longer than width basally; cerci dark brown basally and light distally—***Teloganopsis maculocaudata* (Ikonomov**, **1961)**.

**5(1)** Each of abdominal terga IV–VIII bears a pair of submedian tubercles—**6**.

– All abdominal terga without submedian tubercles, but ridges may be present—**9**.

**6(5)** Head with a pair of occipital protuberances—**7**.

– Head without a pair of occipital protuberances—**8**.

**7(6)** Prothorax with anterolateral projections; transverse irregular row of stout setae on forefemur consists of several short and apically rounded stout setae—***Quatica euphratica* (Kazanci**, **1987)**.

– Prothorax without anterolateral projections; transverse irregular row of stout setae on forefemur consists of a few elongated and slender stout setae—***Serratella karia* (Kazanci**, **1990)**.

**8(6)** Dorsal surface of head, thorax, middle and hind femora covered with small bubble-like setae. Each of abdominal terga I–VIII with a pair of distinct submedian tubercles; only on tergum I the smallest, knob-like, all submedian tubercles bear numerous, mainly elongated, apically rounded, stout setae; caudal filaments and legs are unicolored yellow; coloration of terga III–IX similar, yellow, with brown transverse elongated spots that do not touch each other; terga II and III with transverse brown band; tergum X with indistinct pair of smudged submedian spots—***Serratella leonidi* Martynov & Palatov**, **sp. nov**.

– Dorsal surface of head, thorax and femora without bubble-like setae. Distinct elongated submedian tubercles present on terga IV–VII (VIII), terga II, III and VIII with knob-like tubercles; tergum VIII with occasionally well-developed tubercles; within submedian tubercles only those of terga IV–VIII had more than several short, apically rounded, stout setae; legs with a series of brown slings; apices of caudal filaments brown, basal part with alternating pairs of brown and yellow segments, yellow centrally; abdominal terga dark, without distinct coloration pattern, terga IV–VI and X lighter than other terga—***Serratella ignita* (Poda**, **1761)**.

**9(5)** Head with a pair of occipital protuberances, sometimes indistinct; prothorax with anterolateral projections—***Quatica ikonomovi* (Puthz**, **1971)**.

– Head without a pair of occipital protuberances; prothorax without anterolateral projections—**10**.

**10(9)** Dorsal surface of forefemur with distinct transverse row of about 15 or more extremely long, stout hair-like setae pointed apically. Gill III narrowed—***Torleya major* (Klapálek**, **1905)**.

– Dorsal surface of forefemur without distinct transverse row; several elongated, stout setae rounded or pointed apically, irregularly scattered on dorsal surface. Gill III not narrowed—**11**.

**11(10)** Lateral areas of posterior margin of tergum VIII with long stout setae pointed or apically rounded. Pronotum and occasionally dorsal surface of femora with small, robust, rounded stout setae (these small setae often almost absent)—***Torleya elissa* (Jacobus**, **Zhou & McCafferty**, **2009) comb. nov.**

– Lateral areas of posterior margin of tergum VIII with extremely long, stout setae pointed apically and long, thin, stout hair-like setae. Pronotum and dorsal surface of femora with numerous relatively large, robust, rounded, stout setae—***Torleya* sp. Cauc1**.

It should be noted that almost all of the species, excluding *S. ignita*, *Q. ikonomovi*, *T. major*, *T. mesoleuca* and *T. maculocaudata*, are locally distributed and their distributions do not overlap (Table 3). *Torleya elissa* is relatively widely distributed within the Iranian Plateau, within central and southern parts (Zagros Range) and the northern part (Alborz Range). There is a high probability of finding this species within the Talish Mountains. *Serratella leonidi* Martynov & Palatov **sp. nov.** was found only within the upper part of the Amu Darya river basin in Central Asia. *Serratella karia* is locally distributed within south-western Anatolia, and *Quatica euphratica* (Kazanci, 1987) in the eastern part of Anatolia, where it is found within the upper part of the Euphrates River basin. If there are thorough investigations of target regions, similar local distribution can be evidence of relict nature and the gradual extinction of the group [46]. The unusually stretched (more than 1600 km) but obviously fragmented distribution of *Teloganopsis subsolana* (Allen, 1973) was registered in the Kabul River (Hindu Kush Mountain Range) and in streams within northern Iran (Mazandaran Province).

### 4.2. Notes on Separation of Serratella Edmunds, 1959, Torleya Lestage, 1917 and Quatica Jacobus & McCafferty, 2008

*Serratella* and *Quatica* are related and are possibly polyphyletic genera. Some of their representatives are morphologically similar at the larval stage. The most recent detailed combined molecular and morphological analysis by Ogden et al. [31] did not include any species of *Quatica*. Ubero-Pascal & Sartori [35] included *Quatica* species in their phylogenetic analysis, and they considered *Q. paradinasi* and *Q. ikonomovi* as closely related forms characterized by three characteristics related to larval and egg morphologies: the presence of tubercles and star-like setae in the head and furrows demarcating the mesh units of chorion sculpturing. They did not include *Q. euphratica* in their analysis. The strong support of this clade (Figure 1 in [35]) led them to confirm that *Q. paradinasi* and *Q. ikonomovi* form a monophyletic group belonging to the genus *Quatica*. It is notable that these characteristics are a part of the main distinguishing characteristics of larval section of *Quatica* classification (see [2]). However, at least some larvae of *Q. ikonomovi* lack the star-like setae on head protuberances, based on specimens from Bosnia and Herzegovina which were examined. In these specimens only several short setae with divergent margins and feathered apices are present on the protuberances. Moreover, some of *Serratella* species, e.g., *S. zapekinae* Bajkova, 1967 also have head protuberances that bear similar types of setae. At the same time, *S. zapekinae* distinctly differs from *Quatica* by the shape of the imago male genitalia (Figure 5 in [47]) and structure of the egg chorion (Figures 30–32 in [48]). Therefore, there is currently no set of only larval unique modality of morphological characters that are invariably characteristic for *Quatica* and absent in *Serratella* species. For now, only one character modality of *Quatica* does not occur in all known *Serratella* representatives, namely the presence of anterolateral projections on the pronotum. This characteristic is mentioned in *Quatica* classification by Jacobus & McCafferty [2]. *Quatica* appears to be restricted to the Mediterranean areas [49]; *Serratella* representatives also occur within this region.

The genus *Quatica* requires further study, especially considering the fact that Jacobus & McCafferty ([2]: Figure 99) united a polytomy to create its present composition. According to our phylogenetic reconstruction based on the COI gene, *Quatica* (sequences of the type species *Q. ikonomovi,* were used) is closely related to *Torleya* (including the type species *T. major*), and any significant division of these genera was not observed (Figure 12 and Figure 13). Notably, some of the examined larvae of *Q. ikonomovi* (from Bosnia and Herzegovina) lack the distinct paired median projections on abdominal terga and star-like setae, and only because of prominent anterolateral projections of pronotum, it cannot be placed in the *Torleya* genus. At the same time, some species of *Torleya*, e.g., *T. major* and *Torleya* sp. Cauc1, bear numerous large, feathered, rounded, stout setae that looks like bulbous star-like setae (see Figure 36 in Jacobus et al. [6]).

The genus *Serratella* is also related to *Torleya*. Both genera have no distinct differences at all stages, and were therefore considered as one taxon by some specialists, e.g., as taxon *Torleya*/g2 in Kluge [34]. We noted that the type species of *Serratella* has well-developed, paired abdominal projections, and non-operculate gills; the type species of *Torleya* has abdominal terga without projections, and operculate gills; the genera or subgenera of *Torleya* and *Serratella* are often separated based on one or both of these characteristics, but such taxa appear to be artificial. It should be noted that the *Torleya* type species *T. major* has indistinct operculate gills, so there is only one characters for separation left. At the same time, according to molecular and combined (based on molecular and morphological data) studies, *Torleya* and *Serratella* are spaced apart (see [16,31]). Unfortunately, only several species of these taxa were used in the studies. Our ML tree (Figure 12 and Figure 13) shows the close relation of sequenced *Torleya* species and dispersion of *Serratella* representatives.

On our opinion, the main goal of the future investigations of the genera is to review them based on molecular and morphological data using as many species as possible. *Torleya* representatives with and without distal palisade of denticles on tarsal claw, with and without maxillary palp mandatory, and with and without projections on abdominal terga should be used in future studies.

The winged stages for a significant number of species from the target regions and the presence of cryptic species are still undescribed, together with a lack of data on regional species distribution, and uncertain composition and boundaries of the genera *Serratella*, *Torleya* and *Quatica* show a perspectivity and necessity for the further study of the tribe Hyrtanellini in general, and especially within Western and Central Asia.

## Figures and Tables

**Table 1:** Codes and origin of new sequences used in molecular study.

Species	Voucher Catalogue Number	Locality and Collector	Latitude	Longitude	Date	GenBank ID	GenSeq Nomenclature (Following Chakrabarty et al. [16])
*Serratella leonidi* **sp. nov.**	***Tj6Sersp/5***	Tajikistan, Gorno-Badakhshan Autonomous Province, Ishkoshim district, near Kozideh kishlak (village), Pyandzh River, Palatov D.M. leg.	37.002528	71.467250	29.vi. 2016	OQ077690	genseq-2 COI
*Serratella leonidi* **sp. nov.**	***Tj6Sersp/6***					OQ077691	genseq-2 COI
*Serratella leonidi* **sp. nov.**	***Tj6Sersp/7***					OQ077692	genseq-2 COI
*Serratella ignita*	***Don421Serig/1***	Ukraine, Donetsk Region, vicinity of Debaltseve town, border of Donetsk and Luhansk Regions, Bulavina River, Martynov A.V. leg.	48.315440	38.437849	8.vii. 2011	OQ077686	genseq-4 COI
*Serratella ignita*	***Don421Serig/2***					OQ077687	genseq-4 COI
*Serratella ignita*	***Zkp5Serig/1***	Ukraine, Zakarpattia region, Uzhhorod district, territory of Sil’tse village, Irshava River, Martynov A.V. leg.	48.287250	22.997417	11.v. 2017	OQ077676	genseq-4 COI
*Serratella ignita*	***Bul1Serig/1***	Bulgaria, near Bolgarchevo village, bridge, Strumna River, Godunko R.J. leg.	42.028160	23.042721	21.vii. 2017	OQ077671	genseq-4 COI
*Serratella ignita*	***Bul1Serig/2***					OQ077672	genseq-4 COI
*Serratella ignita*	***Bul1Serig/3***					OQ077673	genseq-4 COI
*Serratella ignita*	***Tur4Serig/1***	Turkey, Dipsiz Önü Stream, Gemicıler village, river, 500 m upstream from the village, in forest near Inebolu–Ayancık road, h—50 m a.s.l., Godunko R.J. leg.	41.960661	33.883996	25.vii. 2017	OQ077677	genseq-4 COI
*Serratella ignita*	***Tur4Serig/2***					OQ077678	genseq-4 COI
*Serratella ignita*	***Tur4Serig/3***					OQ077679	genseq-4 COI
*Serratella ignita*	***Tur4Serig/4***					OQ077680	genseq-4 COI
*Serratella ignita*	***Bul2Serig/1***	Bulgaria, Burgas Region Strandzha Mts., Brodilovo village, Veleka River, bridge, Godunko R.J. leg.	42.081533	27.860061	1.viii. 2017	OQ077674	genseq-4 COI
*Serratella ignita*	***Bul2Serig/2***					OQ077675	genseq-4 COI
*Serratella ignita*	***Arm11Serig/1***	Armenia, Gegharkunik Province, vicinity of Tovaguh village, Dzknaget.	40.618889	44.950833	12.vii. 2015	OQ077681	genseq-4 COI
*Serratella ignita*	***Arm9Serig/1***	Armenia, Kotayk Province, vicinity of Bjni village, Hrazdan River, 40°27′51″ N 44°40′25″ E, h ~ 1497 m a.s.l., Martynov A.V. leg.	40.464167	44.673611	11.vii. 2015	OQ077682	genseq-4 COI
*Serratella ignita*	***RF18Sersp/1***	Russian Federation, Krasnodar krai, Khosta district, Khosta River (at the mouth), Palatov D.M. leg.	43.508183	39.868783	26.iv. 2015	OQ077684	genseq-4 COI
*Serratella ignita*	***RF18Sersp/2***					OQ077685	genseq-4 COI
*Serratella ignita*	***RF27Serig/2***	Russian Federation, Murmansk region, Kolsky District, Teriberka River, near the highway bridge between the villages of Teriberka and Dalniye Zelentsy, Palatov D.M. leg.	68.883176	34.351521	30.vii. 2015	OQ077669	genseq-4 COI
*Serratella ignita*	***RF22Serig/2***	Russian Federation, Tula Region, Suvorovsky District, Oka River, near mouth of the Svobod’ River, Palatov D.M. leg.	54.192120	36.322644	10.vi. 2015	OQ077670	genseq-4 COI
*Serratella ignita*	***RF21Serig/2***	Russian Federation, Republic of Karelia, Pudozhsky District, Tuba river, about 10 km S from village of Pudozhgorsky, Palatov D.M. leg.	62.246913	35.832382	22.vii. 2015	OQ077688	genseq-4 COI
*Serratella ignita*	***Iran8Sersp3/1***	Iran, Lorestan Province, Tenge-Kholeven River, upstream of the Cham Chit village, Palatov D.M. leg.	33.379546	48.973726	29.vi. 2019	OQ077683	genseq-4 COI
*Serratella zapekinae*	***RF29Serzap***	Russian Federation, Khabarovsk Krai, Nanaysky district, Tormasy River, 1 km from the mouth, Vorob’yeva L. leg.	49.339239	137.573866	9.viii. 2016	OQ077689	genseq-4 COI
*Torleya elissa*	***Iran4Serel/4***	Iran, Chaharmahal and Bakhtiari Province, left tributary of Zayandeh Rud, NE of Dimeh, Bojková J., Soldán T., Imanpour Namin J. leg.	32.519300	50.227333	1.v. 2016	OQ077700	genseq-2 COI
*Torleya elissa*	***Iran4Serel/5***					OQ077701	genseq-2 COI
*Torleya elissa*	***Iran8Sersp1/1***	Iran, Lorestan Province, Tenge-Kholeven River, upstream of the Cham Chit village, Palatov D.M. leg.	33.379546	48.973726	29.vi. 2019	OQ077698	genseq-2 COI
*Torleya elissa*	***Iran9Sersp1/1***	Iran, Lorestan Province, Dare Daei River—left tributaries of the Tenge-Gendumer-Darre-Luku River, Palatov D.M. leg.	33.186067	49.510117	13.vi. 2019	OQ077699	genseq-2 COI
*Torleya major*	***Zkp6Tormaj/1***	Ukraine, Zakarpattia region, Vil’khovatyi district, Tysa River, Martynov A.V. leg.	48.025117	24.168117	12.v. 2017	OQ077702	genseq-4 COI
*Torleya major*	***Zkp6Tormaj/2***					OQ077706	genseq-4 COI
*Torleya major*	***Zkp7Tormaj/1***	Ukraine, Zakarpattia region, Rakhiv district, territory of Bilyn village, Tysa River, Martynov A.V. leg.	48.115833	24.265150	12.v. 2017	OQ077703	genseq-4 COI
*Torleya major*	***Zkp7Tormaj/2***					OQ077704	genseq-4 COI
*Torleya major*	***Zkp7Tormaj/3***					OQ077705	genseq-4 COI
*Torleya* sp. Cauc1	***RF17Torsp/3***	Russian Federation, Krasnodar krai, Sochi urban okrug, Khosta district, vicinity of Illarionovka village, Malaya Khosta River, Palatov D.M. leg.	43.582917	39.872900	25.iv. 2015	OQ077697	genseq-4 COI
*Torleya* sp. Cauc1	***RF17Torsp/2***					OQ077696	genseq-4 COI
*Quatica ikonomovi*	***BH1Epliko/3***	Bosnia and Herzegovina, Jala River W of Tuzla town, bridge, Godunko R.J. leg.	44.516818	18.588041	14.vii. 2016	OQ077693	genseq-4 COI
*Quatica ikonomovi*	***BH1Epliko/4***					OQ077694	genseq-4 COI
*Quatica ikonomovi*	***BH1Epliko/5***					OQ077695	genseq-4 COI
*Ephemerella mucronata*	***Zkp6Eplmuc/2***	Ukraine, Zakarpattia region, Vil’khovatyi district, Tysa River, Martynov A.V. leg.	48.025117	24.168117	12.v. 2017	OQ077707	genseq-4 COI

**Table 2:** Genetic distances (COI) between sequenced Hyrtanellini species, which occur in Western and Central Asia, and within the species calculated using the Kimura 2-parameter (K2) model and Tamura 3-parameter (T92) model with a gamma distribution (G) (K2/T92+G). N—number of used sequences.

	*Serratella ignita*	*Serralella leonidi* sp. nov.	*Quatica ikonomovi*	*Torleya* sp. Cauc1	*Torleya elissa*	*Torleya major*
***Serratella ignita***	0.065/0.116 n = 109					
***Serralella leonidi* sp. nov.**	0.207/0.675	0.003/0.003 n = 3				
***Quatica ikonomovi***	0.195/0.584	0.213/0.698	0.071/0.132 n = 10			
***Torleya sp.* Cauc1**	0.207/0.666	0.216/0.708	0.209/0.688	0.005/0.005 n = 2		
***Torleya elissa***	0.204/0.658	0.227/0.812	0.183/0.520	0.148/0.349	0.013/0.014 n = 4	
***Torleya major***	0.238/0.917	0.232/0.836	0.217/0.739	0.216/0.755	0.223/0.867	0.072/0.143 n = 19

**Table 3:** Distribution of Hyrtanellini Allen, 1980 in Western and Central Asia.

Species	Western Asia																			Central Asia					
	TR	SY	LB	PS	IL	EG *	JO *	SA *	YE *	OM *	AE *	QA *	BH *	KW *	IQ	IR	AZ	AM	GE	TM	AF	TJ	KG	UZ	KZ
*Serratella ignita* (Poda, 1761)	●	●													●		●	●	●						●
*Serratella karia* (Kazanci, 1990)	●																								
*Serratella leonidi* Martynov & Palatov, **sp. nov.**																					●	●			
*Torleya elissa* (Jacobus, Zhou & McCafferty, 2009)																●									
*Torleya major* (Klapálek, 1905)	●														●										
*Quatica euphratica* (Kazanci, 1987)	●																								
*Quatica ikonomovi* (Puthz, 1971)		●																							
*Teloganopsis bauernfeindi* (Thomas, Marie and Dia [in Marie, Dia and Thomas], 1999)		●	●																						
*Teloganopsis maculocaudata* (Ikonomov, 1961)																●									
*Teloganopsis mesoleuca* (Brauer, 1857)	●		●		●																				
*Teloganopsis subsolana* (Allen, 1973)																●					●				

*Western Asia*: *—There are no reliable published data about the diversity and distribution of tribe Hyrtanellini in Palestinian territories (PS), Egypt (EG), Jordan (JO), Saudi Arabia (SA), Yemen (YE), Oman (OM), United Arab Emirates (AE), Qatar (QA), Bahrain (BH) and Kuwait (KW). TR—Turkey (based on summarized data published by Kazanci and Türkmen [37] and Salur et al. [7]; Kazanci [38]: original description of *Quatica euphratica* based on larvae from Eastern Anatolia; Kazanci [4]: original description of *Serratella karia* based on larvae from Western Anatolia); SY—Syria (based on Koch [39] and Marie et al. [11]); LB—Lebanon (based on Marie et al. [11]; original description of *Teloganopsis bauernfeindi* (Thomas, Marie and Dia, 1999) [in Marie, Dia and Thomas] based on larvae from Orontes River basin); IL—Israel (based on Sartori [5]); IQ—Iraq (based on Al-Zubaidi et al. [40] and Khudhur & Sroka [9]); IR—Iran (based on summarized data published by Bojková et al. [8]); AZ—Azerbaijan (based on Kasymov [41]: *S. ignita* reported from Nizami and Shusha Districts; *Torleya* sp. was also reported from Agdam District. Palatov & Sokolova [42] that recorded *Torleya* cf. *major* from Talysh Mts—it is highly possible that it belongs to undescribed species which were marked here by us as *Torleya* sp. Cauc1); AM—Armenia (based on summarized data published by Hrivniak et al. [43]); GE—Georgia (based on summarized data published by Gabelashvili et al. [44]). *Central Asia*: There are no reliable published data about the diversity and distribution of tribe Hyrtanellini in Turkmenistan (TM), Kyrgyzstan (KG) and Uzbekistan (UZ). AF—Afghanistan (Allen [10]: original description of *Teloganopsis subsolana* based on larvae from the Kabul Riv.; *Serratella leonidi* Martynov & Palatov, **sp. nov** described in this article from the border of Tajikistan and Afghanistan); TJ—Tajikistan (*Serratella leonidi* Martynov & Palatov, **sp. nov.**, described in the present paper based on larvae from the Kofarnihon and Pyandzh Rivs); KZ—Kazakhstan (based on Smirnova [45]).

## Data Availability

All data are available in the paper.
